# Lung nodules and IgG4 related disease: a single-center based experience

**DOI:** 10.1186/s12890-020-01250-3

**Published:** 2020-08-14

**Authors:** Yan Xie, Anji Xiong, Tony Marion, Yi Liu

**Affiliations:** 1 Department of Rheumatology and Immunology, West China Hospital, Sichuan University, Chengdu, China; 2 Department of Microbiology, Immunology, and Biochemistry, University of Tennessee Health Science Center, Memphis, TN USA

**Keywords:** Clinical characteristics, IgG4 related disease, IgG4 related lung disease, Lung nodules, Radiological characteristics

## Abstract

**Background:**

This study was undertaken in an attempt to characterize the frequency and clinical features of lung nodules in IgG4 related disease (IgG4-RD) patients as an insight for help with the diagnosis of lung nodules.

**Methods:**

A retrospective study was carried out in West China Hospital, Sichuan University from January 2012 to December 2018, 89 patients with definite IgG4-RD were enrolled.

**Results:**

Fifty of 89 patients with definite IgG4-RD had radiologically confirmed lung nodules, 6 of whom were diagnosed with definite IgG4 related lung disease. Lung nodules detected in more than 40 patients were small and solid, always with regular margins. Multiple (41/50) and bilateral (34/50) distributions was also a major characteristic of these lung nodules. Lobulation and speculation were simultaneously detected in 3 patients, including 2 patients combined with pleural indentation. Calcification of nodules was detected in only one patient. Thirty-seven patients also had additional radiological abnormalities of lungs, including ground-glass opacity (21/50), thickening of pleura (9/50), thickening of interlobular septa (4/50), thickening of bronchial wall (3/50), pleural effusion (4/50), mass (3/50), interstitial changes (5/50), and mediastinal or hilar lymphadenopathy (32/50). Most patients (44/50) were treated with glucocorticoids alone or combined with immunosuppressive agents. Sixteen patients received a re-examination by chest computed tomography (CT) scan after treatment, 10 of whom showed a decrease in the size and/or the number of nodules.

**Conclusions:**

The incidence of lung nodules in IgG4-RD patients can be high. For an IgG4-RD patient with lung nodules, the possibility that the lung nodules related to IgG4-RLD is high. It is hard to differentiate IgG4 related lung nodules from other lung diseases, in particular, lung cancer. Radiological characteristics and positive responses to glucocorticoids and immunosuppressive agents can help with the differential diagnosis. For these patients, regular follow-up is also important.

## Background

Clinical presentation of nodules in the lungs of patients can be a challenge for diagnosis, especially when the nodules are small and asymptomatic. The nodules may be difficult to biopsy for histopathology because of size and location, for example, very small nodules deeply imbedded in the lung. The first diagnosis will likely be some form of neoplasm. When that diagnosis is determined to be incorrect, many physicians, especially respiratory physicians and thoracic surgeons, may be unsure of the next step toward diagnosis and treatment. Affected patients, of course, will be anxious for a confirmed diagnosis since they will likely assume the small nodules are an early stage of lung cancer. A recently recognized disease, immunoglobulin G4-related disease (IgG4-RD), must be considered for such presentation of asymptomatic small nodules, particularly in the lungs, liver, or lymph nodes [1].

IgG4-RD is a chronic, fibrotic inflammation characterized by the involvement of multiple organs. The most common manifestations of the disease include swelling of salivary and lacrimal glands, lymphadenopathy, and type 1 autoimmune pancreatitis (AIP). Other organs, such as lung, bronchi, kidney, retroperitoneum, thyroid, heart, mesenterium, meninges, and skin, can also be involved. Frequently, but not always, serum IgG4 levels will be elevated [2]. IgG4 is the rarest of the IgG subclasses, generally has relatively low antigen affinity, and is unable to bind complement component 1q (C1q) and activate the complement cascade [3]. IgG4 can exchange Fab arms by swapping a heavy chain and attached light chain with a heavy-light chain pair from another IgG4 molecule, which provides the basis for the anti-inflammatory activity attributed to IgG4 antibodies [4] A key pathological feature of IgG4-RD nodules is a dense lymphoplasmacytic infiltrate organized into a storiform pattern, which frequently forms a tumefactive mass that may destroy an involved organ [1]. When the tumefactive mass occurs in the lungs, it may present as a nodule or ground-glass opacity on radiology [5]. IgG4-related lung disease (IgG4-RLD) is the lung involvement of IgG4-RD, which was first described in 2004 in a patient with interstitial pneumonia, autoimmune pancreatitis and IgG4-positive plasma cells in the interstitium [6]. The IgG4-RLD presentation can be heterogeneous, and its radiologic manifestations are often extensive, even when clinically asymptomatic [7].

Although lung nodules have been described in 31 to 46% of IgG4-RLD in a few case reports and reviews to date [5, 8, 9], no study has employed a systematic analysis of IgG4-RD patients with lung nodules. Here we present retrospective data of the clinical and radiological features of IgG4-RD patients from a single medical center’s experience. Our goal is to provide clinical information and insight for the diagnosis of IgG4-RD in patients who present with asymptomatic, non-neoplastic small lung nodules.

## Methods

### Patients information

Patients with definitive IgG4-RD were selected from all departments of West China Hospital, Sichuan University from January 2012 to December 2018. The clinical, serological, and radiographic imaging characteristics and treatment responses of the patients were analyzed.

Our study was performed according to the rules of the hospital’s medical ethics committee. Informed consent was obtained in accordance with the institutional guidelines.

### Diagnosis of IgG4-RD

The diagnosis of IgG4-RD was made according to the comprehensive diagnostic criteria for IgG4-RD published in 2011 by Umdehara et al. [10] The criteria for definite, probable, and possible IgG4-RD are as follows:

Definite:1 + 2 + 3

Probable:1 + 3

Possible:1 + 2

1. Diffuse or localized swelling or masses in single or multiple organs.
2. Elevated serum IgG4 > 135 mg/dl.
3. Histopathology of biopsied nodules shows:
  (a) Marked lymphocyte and plasmacyte infiltration and fibrosis
  (b) Infiltration of IgG4 plasma cells defined as > 40% IgG4 plasma among all IgG plasma cells and > 10 IgG4 plasma cells per 40X field.

### Chest CT protocols

CT images were available for all patients involved in this study. Because of the retrospective nature of this study, chest CT scanning protocols were varied. The scanning was performed on one of the six machines ranging from 16-detector to 128-detector CT scanners (Philips Medical Systems, Best, the Netherlands or Siemens Medical Systems, Erlangen, Germany), with patients in the supine position. For patients who underwent contrast-enhanced examination, an intravenous nonionic contrast medium (Iopamidol, 350 mg/ml, 80-100 ml) was given and imaging started 25 s later after injection. To minimize motion artifacts, CT images were acquired during a single breath-hold. The main parameters were shown as follows: tube voltage = 100-120kv, tube current = 70-200 mA, slice thickness = 5 mm, section interval = 5 mm.

### Definition of lung nodules and IgG4-RLD

CT images of patients’ chests were analyzed by radiologists. The lung nodule(s) were defined as rounded or irregular opacity, well or poorly defined, measuring up to 3.0 cm in diameter [11]. Nodules were categorized as large or small as follows: large, 1 cm ≤ diameter ≤ 3.0 cm or small, diameter < 1 cm. If the diameter of the lung lesion is larger than 3.0 cm in the CT images, the lesion was defined as a mass.

IgG4-RLD is defined as IgG4-RD, diagnosed as above, that includes diffuse or localized swelling or masses in one or both lobes of the lungs with histopathology that includes marked lymphocyte and plasmacyte infiltration and fibrosis and/or infiltration of IgG4 plasma cells defined as > 40% IgG4 plasma among all IgG plasma cells and > 10 IgG4 plasma cells per 40 × field.

### Statistics

Statistical analyses were performed with GraphPad Prism, version 6 (GraphPad Software Inc., La Jolla, CA, USA). Descriptive data are reported as median (interquartile range) and frequencies are percentages. Continuous variables without normal distribution were expressed as median and interquartile (IQR). For comparison between groups, the unpaired Student’s t-test was used for continuous variables and chi-square test was used for categorical variables. All statistical tests were 2-sided, and results with *P* values< 0.05 were considered statistically significant.

## Results

From January 2012 to December 2018, a total of 89 patients were diagnosed with definitive IgG4-RD in West China Hospital, Sichuan University, 50 (56%) of whom had CT-confirmed lung nodules were included in this retrospective study.

### Patient description and clinical presentation

Demographic and clinical data for all patients are summarized in Table 1. The median age of the patients with lung nodules was 60 (48–66) years at diagnosis with a male: female ratio of 39:11. Almost half of the patients had their initial diagnosis of IgG4-RD in the Department of Rheumatology. The duration of symptoms before diagnosis averaged 20 months (range 1–240 months). Forty-two of the 50 patients had no pulmonary infection or a history of cancer and other chronic pulmonary diseases. And more than half of the patients (29/50) had a history of smoking.

**Table 1:** Clinical characteristics of IgG4-RD patients

Parameters	Patients with lung nodules(*n* = 50)	Patients without lung nodules(*n* = 39)	*P* value
Age, median(IQR), years	60(48–66)	53(40–72)	0.30
Men/women, n (%)	3.55:1(39:11)	2.25:1(27:12)	0.35
Smoking history, n (%)	29(58.0)	13(33.3)	0.02*
Duration of disease, median(IQR), months	7(3–12)	5(2–13)	0.60
Serum IgG4, median(IQR), mg/dl	1185(426–2163)	735(371–2130)	0.48
Elevated ESR, n (%)	20(40.0)	9(23.1)	0.09
Elevated CRP, n (%)	9(18.0)	9(23.1)	0.55
Reduced C3, n (%)	23(46.0)	12(30.8)	0.14
Reduced C4, n (%)	16(32.0)	7(17.9)	0.13
Positive ANA, n (%)	20(40.0)	18(46.2)	0.56
First symptoms, n (%)			
Salivary gland swelling	8(16.0)	3(7.7)	
Lymphadenopathy	6(12.0)	3(7.7)	
Lacrimal gland swelling	9(18.0)	8(20.5)	
Cough	7(14.0)	2(5.1)	
Jaundice	4(8.0)	2(5.1)	
Dysuria	4(8.0)	1(2.6)	
Fever	2(4.0)	7(17.9)	
Abdominal pain	4(8.0)	8(20.5)	
Gum swelling	1(2.0)	0	
Low back pain	2(2.0)	0	
Edema	3(6.0)	3(7.7)	
Fatigue	0	1(2.6)	
Diarrhea	0	1(2.6)	
Number of extrapulmonary organs involved, median(IQR)	2(1–3)	2(1–3)	0.14
Extrapulmonary organ involvement, n (%)			
Salivary glands(submandibular gland and parotid gland)	21(42.0)	8(20.5)	
Lymph node	19(38.0)	15(38.5)	
Lacrimal gland	14(28.0)	8(20.5)	
Pancreas	15(30.0)	10(25.6)	
Kidney	16(32.0)	8(20.5)	
Liver	12(24.0)	6(15.4)	
Nasal sinus	6(12.0)	5(12.8)	
Skin	2(4.0)	2(5.1)	
Bile ducts	4(8.0)	9(23.1)	
Peritoneum	1(2.0)	2(5.1)	
Pituitary	1(2.0)	0	
Thyroid glands	3(6.0)	1(2.6)	
Gingiva	1(2.0)	0	
Pericardium	1(2.0)	1(2.6)	
Blood	0	2(5.1)	

Data are expressed as median(interqurtile range) for contious variables, and frequencies(percentages) for categorical variables

*IgG4-RD* IgG4 related disease, *ESR* Erythrocyte sedimentation rate, *CRP* C-reactive protein, *C3* Complement 3, *C4* Complement 4, *ANA* Antinuclear antibody, *IQR* Interquartile range

* *P* < 0.05

#### First symptoms

For patients with lung nodules, the most common first symptoms were salivary gland swelling (8/50) and lacrimal gland swelling (9/50). Seven patients presented with cough as their first symptom. There were several rare symptoms including fever, gum swelling, low back pain, and edema.

#### Extrapulmonary organs involved

Most patients had multiple sites or organs involved (median number 2, IQR1–3). Twenty-one patients had at least three extrapulmonary organs or sites affected. The most common involved extrapulmonary organs included salivary gland (21/50), lymph node (19/50), lacrimal gland (14/50), pancreas (15/50), and kidney (16/50). Other sites included liver, nasal sinus, peritoneum, pituitary, skin, thyroid glands, bile ducts, gingiva, and pericardium.

#### Serological characteristics

Serum levels of IgG4, erythrocyte sedimentation rate (ESR), C-reaction protein (CRP), and complements were quantified. Serum IgG4 concentrations were measured in all patients in this study, with a median value of 1185 mg/dl (IQR426–2163) for patients with lung nodules compared to 735 mg/dl (IQR371–2130) for IgG4-RD patients without lung nodules (Table 1). Elevated ESR and serum CRP levels were found in 20 and 9 patients, respectively, among patients with lung nodules versus 9 patients with elevated ESR and 9 patients with elevated CRP among IgG4-RD patients without lung nodules. Twenty-four patients with lung nodules versus 12 without, had reduced serum complement. Twenty patients with lung nodules versus 18 without, had antinuclear antibody (ANA) (> 1:100 was defined as positive). There were no statistically significant differences in serological measurements between IgG4-RD patients with and without lung nodules (Table 1).

#### Comparison of clinical characteristics

The general clinical characteristics of patients with lung nodules were compared with those of patients without lung nodules. As the results indicated in Table 1, significant differences were observed in smoking history between the two groups, while no significant differences in age, gender ratio, duration of symptoms before diagnosis, and the number of extrapulmonary involved organs were observed.

### Radiological characteristics of the lungs

Fifty patients included in this study had lung nodules revealed in chest CT images. The radiological characteristics of lungs among the 50 patients with lung nodules are summarized in Table 2 and Table 3.

**Table 2:** Radiological characteristics of lungs before drug therapy

Patients with indicated lung nodules			n (%)
Size			
Small nodule only		single	7(12.0)
		multiple	38(78.0)
Large nodule only			2(4.0)
Both small and large nodule			3(6.0)
Distribution			
Laterality			
Unilateral			16(32.0)
Bilateral			34(68.0)
Lobe			
Upper lobe			6(12.0)
Middle or lower lobe			12(24.0)
Random			32(64.0)
Type			
Ground-glass nodule only			1(2.0)
Solid nodule only			46(92.0)
Both ground-glass and solid nodule			3(6.0)
Margin			
Irregular or untidy			6(12.0)
Regular			44(88.0)
Second associated features			
Lobulation			3(6.0)
Spiculation			3(6.0)
Pleural retraction			2(4.0)
Calcification			1(2.0)
Changes of nodules after treatments(*n* = 16)			
Smaller or disappear			10(20.0)
No difference			6(12.0)
Other radiological features			
Ground-glass opacity			21(42.0)
Thickening of pleura			9(18.0)
Thickening of interlobular septa			4(8.0)
Thickening of bronchial wall			3(6.0)
Pleura effusion			4(8.0)
Mass			3(6.0)
Consolidation			1(2.0)
Interstitial changes			5(6.0)
Mediastinal and hilar lymphadenopathy			32(64.0)

**Table 3:** Radiologic findings in 6 patients with definite IgG4-RLD

Patients No.	Large nodule	Small nodule	Ground-glass opacity	Mass	Thickening of pleura	Thickening of interlobular septa	Pleura effusion	Enlarged mediastinal or hilar lymph node
1	Solitary		Yes		Yes			Yes
2		Multiple	Yes	Yes		Yes	Yes	Yes
3	Solitary		Yes		Yes			Yes
4		Multiple		Yes			Yes	Yes
5		Multiple		Yes				Yes
6		Multiple	Yes		Yes		Yes	Yes

**Fig. 1:**
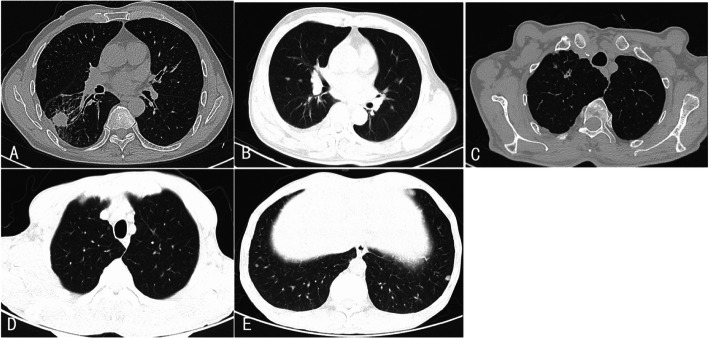
Radiological manifestations of lung nodules in IgG-RD patients via chest computed tomography scans. **a** A single large solid nodule, with irregular margin, was detected in the right lung. Thickening of pleura and tracheal traction were also noted. **b** Multiple small and solid lung nodules were scattered in both lungs. **c** A single, small, and solid nodule was shown in the right lung apex. Lobulation, spiculation, and pleural indentation of the nodule can be noted. Thickening of pleura and the bronchial wall can also be observed. **d**/**e** Multiple solid and ground-glass nodules were shown in both lungs

Five patients had large lung nodules (Fig. 1a) with diameters ranging from 1.2 to 2.6 cm, while almost all patients (48/50) showed small lung nodules (Fig. 1b-e). Most patients (41/50) had multiple nodules. Only 7 patients had single, small nodules. For the distribution of lung nodules, 34 patients had bilateral nodules, accounting for more than half of the 50 cases. When considering the pulmonary lobe involvement, the number of patients with nodules located only in the upper lobe and middle or lower lobe was 6 and 12, respectively. The rest 32 patients had nodules randomly distributed in different lobes at the same time.

**Fig. 2:**
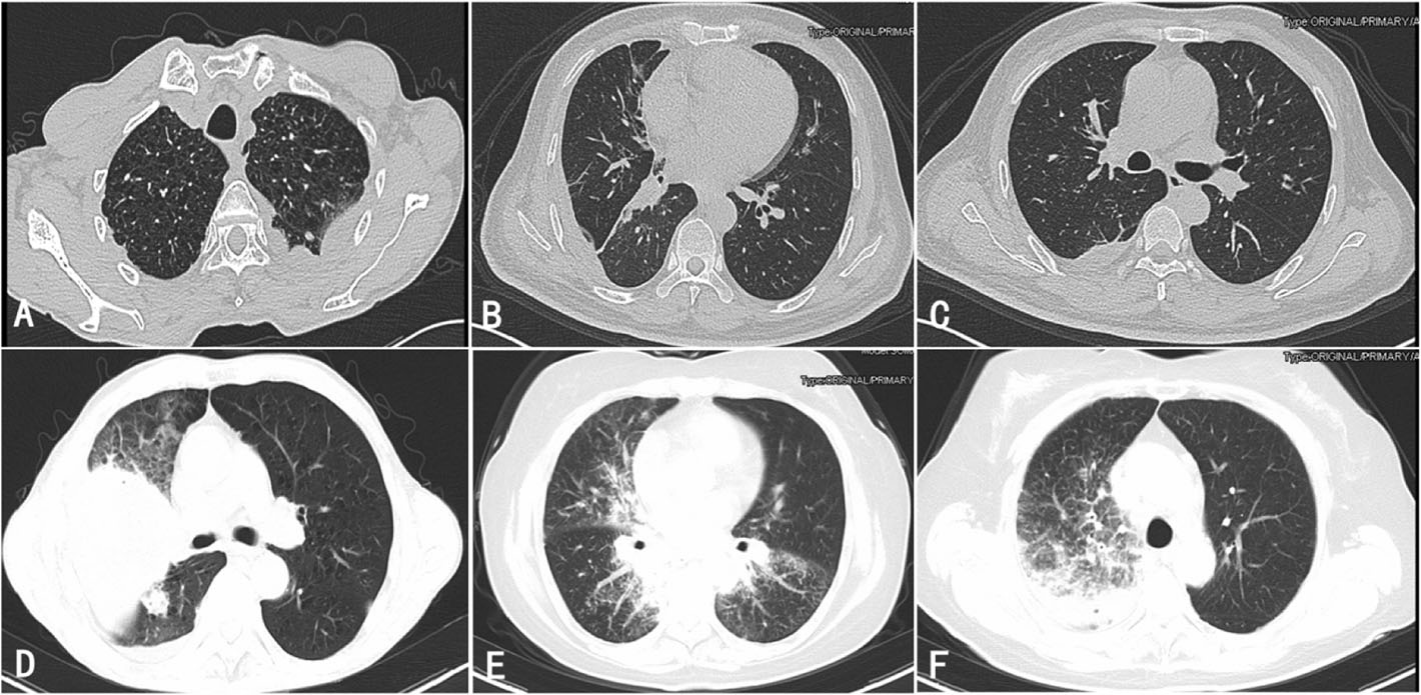
Other radiological manifestations of lungs in IgG-RD patients via chest computed tomography scans. **a** Thickening of the left pleura can be observed. A small nodule near the thickened pleura can also be noted. **b**/**c** Thickening of interlobular septa and pleura effusion in the right lung can be observed. **d** Mass and ground-glass opacity were shown. **e**/**f** Small lung nodules, combined with ground-glass opacity, consolidation, thickening of interlobular septa, and thickening of bronchial wall, can be observed

For the lesion densities on CT, solid nodules can be detected in almost all patients (49/50). Only 4 patients showed ground-glass nodules, all of which were small nodules. Nodules in 3 out of the 4 patients showed both ground-glass and solid patterns (Fig. 1d, Fig. 1e). Most patients (44/50) showed regular and well-defined nodule margins. Only 6 patients had nodules with irregular or poorly defined margins. Some other features of nodules, including lobulation, spiculation, pleural indentation, and calcification, were also explored. All these signs were rare in our study. Lobulation and speculation (Fig. 1c) were simultaneously detected in 3 patients, including 2 patients combined with pleural indentation (Fig. 1c). Calcification of nodules was detected in only one patient. Thirty-seven patients, including the 6 patients with definite IgG4-RLD, showed some other radiological abnormality of lungs (Fig. 2), which included ground-glass opacity(21/50), thickening of pleura(9/50), thickening of interlobular septa (4/50), thickening of bronchial wall(3/50), pleural effusion(4/50), mass(3/50), consolidation(1/50), interstitial changes(5/50), and mediastinal or hilar lymphadenopathy(32/50).

Six patients were diagnosed as definite IgG4-RLD. The chest CT findings of these patients were shown in Table 3. All of the 6 patients showed mixed patterns in CT changes. Two patients had single large lung nodules only, while the rest 4 patients showed multiple small lung nodules only. Mass, thickening of pleura, thickening of interlobular septa, ground-glass opacity, and pleura effusion were detected in 3, 3,1, 4, and 3 patients, respectively. Enlarged mediastinal or hilar lymph node was detected in all IgG4-RLD patients.

### Therapy and responses

The results of therapy and responses are shown in Table 2 and Table 4. For the treatment of the 50 patients with lung nodules, 44 patients received prednisone with or without additional anti-inflammatory or immunosuppressive drugs: 21 with prednisone only, 17 with prednisone and cyclophosphamide, 5 with prednisone and mycophenolate mofetil or methotrexate, and 2 with prednisone and azathioprine. Three patients received a surgical treatment only and 3 patients with mild disease received symptomatic therapy.

**Table 4:** Treatments of IgG4-RD patients

Treatments		Patients with lung nodules n (%)
Pred only		21 (42.0)
Pred+immunosuppressive agents	Pred+CYC	17 (34.0)
	Pred+MMF	2 (4.0)
	Pred+MTX	3 (6.0)
	Pred+AZA	1 (2.0)
others		6 (12.0)

*IgG4-RD* IgG4 related disease, *Pred* prednisone, *CYC* cyclophosphamide, *MMF* mycophenolate mofetil, *MTX* methotrexate, *AZA* azathioprine; Others, surgical treatment or symptomatic, no coticosteriod or immunosuppressive agents was given

Most patients (48/50) improved to some degree after treatments during the duration of hospitalization. Sixteen patients received a reexamination of chest CT scan after treatment. The durations between CT scans were at least 1 month. Ten patients showed a decrease in the size and/or the number of nodules, while 6 patients showed no difference between pre- and post-therapy CT images.

## Discussion

Lung nodules are small rounded lesions with at least two-thirds of its margins surrounded by lung parenchyma and not associated with atelectasis or lymphadenopathy. In this study, we focused on the lung nodules of IgG4-RD patients and 50 (56%) IgG4-RD patients presented with lung nodules in CT images. As far as we know, there hasn’t been any study concerned about the incidence of lung nodules in IgG4-RD patients. Previous studies have reported that lung nodules were incidentally identified in approximately 15–30% of the social-demographically population [12, 13]. Our result revealed that the incidence of lung nodules in IgG4-RD patients was much higher.

Comparing the clinical characteristics of patients with lung nodules with that of patients without lung nodules, no significant difference was found in terms of age, gender ratio, duration of symptoms before diagnosis, number of extrapulmonary involved organs and serological characteristics, indicating that these factors have no association with the progress of nodule formation. For clinical symptoms, only 7 patients were observed with cough. Most patients, especially patients with small nodules only, were relatively asymptomatic despite substantial burdens of disease within the lung. Clinical symptoms of lung disease depend on the location and size of lesions and are often nonspecific for the diagnosis of some lung disease including IgG4-RLD.

In our study, six patients were diagnosed as definite IgG4-RLD. All of the 6 patients had lung nodules. Consistent with results of previous studies conducted by Inoue et al [5] and Sun et al [14]*,* this result revealed that nodular lesion can be a common manifestation of IgG4-RLD. Together with lung nodules, some other chest CT findings, including mass, solid nodules, round-shaped glass opacity, thickening of bronchovascular bundles and interlobular septa, alveolar interstitial changes like honeycombing and bronchiectasis, lobar or segmental consolidation, and lymph node enlargement et al, were often seen in IgG4-RLD patients. And these CT changes always present as various mixed patterns [5, 9, 14–18]. In this study, except lung nodules, we also detected mass, ground-glass opacity, thickening of pleura, pleura effusion, mediastinal and hilar lymphadenopathy, and thickening of interlobular septa in IgG4-RLD patients. It is worth noting that IgG4-RLD related nodule lesion reported previously are always single, solid, and large type, together with or without multiple small nodules [5, 9, 14–18]. And these nodules are always solid and with spiculated or irregular margins. In our study, only 2 IgG4-RLD patients had large nodules, while 4 of the 6 IgG4-RLD patients presented as multiple small nodules. And only one of the 6 patients had nodules with irregular margins. As pulmonary biopsy results are hard to get, this inconsistency may be caused by a small sample size of studies related to IgG4-RLD. Thus more studies are needed to clarify the CT imaging features of IgG4-RLD.

As lung nodules could also be caused by infection, malignancy, or some other pulmonary disorder, we cannot be certain that in the absence of definitive nodular biopsies, all of the lung nodules in our study were related to IgG4-RLD. For an IgG4-RD patient with lung nodules, the probability of lung nodules related to IgG4-RLD is very high especially in the absence of other lung diseases. In a study reported by Tsushima et al, 5 of 6 IgG4-RD patients with lung nodules were confirmed to be IgG4-RLD by biopsy [15]. In our study, most patients had no pulmonary infection or a history of cancer and other chronic pulmonary diseases. For these patients, the probability of lung nodules related to IgG4-RLD is very high. Besides, CT findings of our study also revealed that most of the lung nodules in IgG4-RD patients were small and solid, always with regular margins. Multiple and bilateral distributions was also a major characteristic of these lung nodules. Lung nodules in one patient also showed calcification. These radiologic features and distribution of lung nodules were usually regarded as benign [13, 19].

It is noteworthy that three patients with large lung nodules showed signs of lobulation, spiculation, and pleural indentation, all of which may predict an increased risk of malignancy [13, 19]. As radiological findings of IgG4-RLD varied, these signs can often be observed in lung nodules and masses related to IgG4-RLD [5, 14]. Besides, we also found that the proportion of lung nodules in smokers was significantly higher than that in non-smokers, indicating that smoking may be a risk factor of these lung nodules. The proportion of elderly in IgG4-RD patients was not small. Therefore, for IgG4-RD patients with lung nodules, it’s necessary but hard to differentiate IgG4-RLD from lung cancer. As mentioned above, the simultaneous presence of other kinds of lung lesions may provide support for the diagnosis of IgG4-RLD and help with the differential diagnosis. In our study, most (37/50) patients also had the simultaneous presence of other CT findings, including thickening of pleura, thickening of interlobular septa, thickening of bronchial wall, pleural effusion, presence of an undefined mass, interstitial changes, consolidation, ground-glass opacity and mediastinal or hilar lymphadenopathy, which may help to increase the possibility of IgG4-RLD.

Most IgG4-RLD patients have a significant response to glucocorticoid therapy, which can help to further distinguish IgG4-RD nodules and malignancy [20]. In our study, 16 patients went through a re-examination of CT scan after glucocorticoid therapy with or without immunosuppressive agents. Ten patients, including 2 patients that had large lung nodules with signs of lobulation, spiculation, pleural indentation, and cavity, showed positive responses. A decrease in the size and/or the number of nodules was observed, which helped to further support the diagnosis of IgG4-RLD.

Sometimes during a routine medical examination, patients may present with lung nodules detected without other pulmonary symptoms. Since nodules may resemble bronchoalveolar carcinoma and raise suspicion of malignancy [4], case reports indicate that patients with this type of lung lesions may have undergone wedge resection or lobectomy for suspected malignancy only to discover upon tissue examination that the relevant nodules were a consequence of IgG4-RD [21, 22]. In our study, a patient with bilateral small lung nodules was first misdiagnosed as tuberculosis and another patient with a single large nodule underwent a middle lobectomy and histological findings were consistent with IgG4-RD. Thus, an understanding of nodules related to IgG4-RD is noteworthy. Since IgG4-RD may be a multi-organ disease, the involvement of other organs and serological change can help with diagnosis in patients with undiagnosed lung nodules. In our study, only 6 patients had the pathological proof of IgG4-RLD, while the other patients were diagnosed with IgG4-RD primarily because of clinical manifestations in other organs, particularly salivary glands, lymph nodes, lacrimal gland and pancreas, which is consistent with other studies [16]. In Sun’s study of biopsy-proven IgG4-RLD patients, extrapulmonary involvement was proven in only 1 patient with uveitis mastoiditis. Since patients with only lung involvement are more prone to receive a lung biopsy, the uncommon extrapulmonary involvement may be caused by selection bias [14]. Once the diagnosis of IgG-RD is confirmed, the radiological characteristic and treatment response can help with the diagnosis of IgG4-RLD, thus a regular follow-up is important for these patients.

Our study had some limitations. First, most of the lung nodules involved in our study were not histopathologically proven. Therefore, we were unable to clarify the exact proportion of lung nodules related to IgG4-RLD. Second, due to the retrospective features of our study, the scan protocols of CT varied, and the number of patients with a re-examination of CT was small, which may further limit the value of our study.

## Conclusions

The incidence of lung nodules in IgG4-RD patients can be high. For an IgG4-RD patient with lung nodules, the possibility that the lung nodules related to IgG4-RLD is high. It is hard to differentiate IgG4 related lung nodules from other lung diseases, in particular, lung cancer. Radiological characteristics and positive responses to glucocorticoids and immunosuppressive agents can help with the differential diagnosis. For these patients, regular follow-up is also important.

## Data Availability

All relevant data in this study are freely available to any scientist wishing to use them for non-commercial purposes, without breaching participant confidentiality. And relevant data can be obtained by contacting the corresponding author.

## Abbreviations

IgG4-RD: IgG4 related disease
AIP: Autoimmune pancreatitis
C1q: Component 1q
IgG4-RLD: IgG4-related lung disease
CT: Computer tomographic
IQR: Interquartile range
ESR: Erythrocyte sedimentation rate
CRP: C-reaction protein
ANA: Antinuclear antibody
C3: Complement 3
C4: Complement 4
Pred: Prednisone
CYC: Cyclophosphamide
MMF: Mycophenolate mofetil
MTX: Methotrexate
AZA: Azathioprine
